# A robust, low-temperature, closed-loop anaerobic system for high-solid mixed farm wastes: advancing agricultural waste management solutions in Canada

**DOI:** 10.1007/s11356-024-33654-7

**Published:** 2024-05-23

**Authors:** Vaibhavi Bele, Bernard Goyette, Chunjiang An, Inès Esma Achouri, Oumaima Chaib, Rajinikanth Rajagopal

**Affiliations:** 1 Sherbrooke Research and Development Center, Agriculture and Agri-Food Canada, 2000 College Street, Sherbrooke, QC J1M 0C8 Canada; 2 Department of Building, Civil and Environmental Engineering, Concordia University, Montreal, Quebec H3G 1M8 Canada; 3 Department of Chemical Engineering and Biotechnology Engineering, Université de Sherbrooke, Sherbrooke, QC J1K 2R1 Canada

**Keywords:** Anaerobic digestion, Agricultural multi-substrate, Heavy metal concentrations, Low-temperature, Percolation-recirculation, Methane yield

## Abstract

This study investigates the effectiveness of low-temperature (20 ± 1 °C) anaerobic digestion (AD) for two organic multiple farm substrate combinations: Set 1 comprising chicken manure (CM), dairy manure (DM), and waste corn silage (CS) and Set 2 comprising CM, DM, pig manure (PM), and CS. Inoculum adaptation steps were carried out using CM and CM+DM for Set 1 and Set 2, respectively. Over three consecutive operating cycles spanning 245 days with increasing organic loads, 4.3 and 2.8 g VS L^−1^ d^−1^ for Sets 1 and 2 during Cycles 1 to 5.1 and 4.6 g VS L^−1^ d^−1^for Sets 1 and 2 during Cycle 3, a closed-loop two-stage liquid-solid AD system was employed, with performance assessed via stability ratios of short-chain volatile fatty acids and alkalinity. Results demonstrate that mono-digestion of CM with adapted inoculum yielded the highest biogas production of 424 ± 4 L over 77 days, indicating superior performance by Set 1 during Phase I, whereas a similar performance was observed during Phase 2, where Sets 1 and 2 exhibited highest specific methane yields of 0.233 ± 0.028 and 0.262 ± 0.004 L g^−1^ VS_fed_, respectively, over 68 days. Analysis of heavy metal concentrations in digestates revealed a significant decrease compared to initial raw substrate concentrations, highlighting their role as nutrients for microbial growth. This study, the first of its kind, highlights the potential of low-temperature AD systems to manage diverse organic residues/byproducts and offers insights into effective performance monitoring without compromising system integrity.

**Supplementary Information:**

The online version contains supplementary material available at 10.1007/s11356-024-33654-7.

## Introduction

The intensification of agriculture to meet rising food demand has led to the generation of large quantities of organic waste, including food waste, kitchen waste, crop residues, and livestock effluents, contributing to significant greenhouse gas (GHG) emissions. Despite their prevalence, this organic waste is often neglected, even though it could contribute to the production of renewable energy and thus reduce the impact on the climate if properly treated. Anaerobic digestion (AD) has emerged as one of the biological solutions offering a sustainable, environmentally friendly way of managing organic waste by converting it into valuable resources such as biogas and digestate, while reducing GHG emissions and contributing to efforts to combat climate change (Massé et al. 2014). While AD generally functions optimally at higher temperatures (35–55 °C), the risks of inhibition by free ammonia nitrogen (FAN) simultaneously increase, a phenomenon that can significantly hamper the efficiency of the digestion process. This effect is particularly notable for high-nitrogen wastes such as swine and chicken manures (Rajagopal et al. 2013). Moreover, AD heavily relies on acclimatized inoculum for initiation. In cold climate regions like Canada, the adoption of low-temperature AD or near-psychrophilic AD (15–25 °C) has demonstrated potential in mitigating energy demands related to sustaining digester temperatures (Saady and Massé 2015; Rajagopal et al. 2019; Mahato et al. 2022). It also helps in minimizing inhibition risks arising from FAN, predominantly found in ammonia-rich wastes. However, challenges persist with low-temperature or psychrophilic AD systems, as highlighted in a recent study by Lendormi et al. (2022), which focuses on the difficulties encountered during their start-up and the limited understanding of this initiation phase. This underscores a significant knowledge gap that needs to be addressed to facilitate the development of efficient low-temperature AD systems, particularly suited to small-scale operations in cold regions.

When adapting AD systems to the treatment of agricultural waste, it is essential to take into account the diversity of wastes generally present on farms, such as crop residues, livestock effluents, and bedding, which need to be treated simultaneously. Co-digestion, a method combining different organic wastes, offers a solution to achieve a balanced carbon (C)-to-nitrogen (N) ratio, crucial for efficient AD performance (Shah et al. 2015). This approach not only reduces waste segregation and storage requirements but also promotes a circular economy by utilizing multiple waste streams. In Canada, where more than 35% of farms are oilseed and grain farms and more than 25% are beef and feedlot farms (Statistics Canada 2021), an AD system can effectively treat a variety of agricultural wastes. Such adaptation of AD technology to suit the regional Northern climate and handle mixed farm waste streams underscores its potential in addressing agricultural waste management challenges effectively.

Dry, semi-dry, and wet AD systems are distinguished based on their total solids (TS) content, with wet systems having TS levels up to 10%, semi-dry systems ranging between 10 and 15%, and dry systems exceeding 15%. Wet systems typically require larger digester volumes, leading to increased operational, maintenance, and transportation costs. Conversely, dry AD systems may feature smaller digester volumes; however, inadequate substrate mixing could potentially diminish AD performance due to limited microbe-substrate contact. Therefore, in our recent study (Mahato et al. 2022), a closed-loop, two-stage (solid-liquid) AD system was employed to ensure efficient waste-microbe mixing, enhance mass transfer, and optimize biogas production, rendering it a superior choice for AD processes, particularly for mono-digestion of high-solids chicken manure. Monitoring the performance of the two-stage (solid-liquid) AD system was achieved by assessing the physiochemical parameters of the liquid digesters, which can be sampled more frequently compared to their solid counterparts. Various ratio limits, such as total volatile fatty acids (VFA) to total alkalinity, C3/C2, and (C4+C5)/C2, can be utilized to monitor the stability of the AD process (Khanal 2008; Mahato et al. 2022), with C2–C5 representing acetic, propionic, butyric, and valeric acids, respectively. However, there remains a knowledge gap regarding the treatment of multiple feedstocks using this two-stage AD process, which could prove beneficial for farms generating diverse wastes.

Another critical aspect of AD worth examining is the presence of heavy metals (HMs) in livestock manure and digestates, i.e., before and after the AD process. HMs, commonly found in animal feed additives to promote growth, persist in animal waste, and pose significant challenges to the AD process. While microorganisms involved in AD can utilize low concentrations of HMs for growth (Zhu et al. 2014), elevated HM levels can disrupt microbial activity, hindering biogas production and affecting digestate quality. Certain HMs like copper (Cu) and zinc (Zn) exhibit cytotoxic behavior (Lee et al. 2018), further impacting microbial activity and biochemical reactions. Thus, understanding the presence and behavior of HMs in livestock manure throughout the AD process is essential for optimizing biogas production and ensuring the quality of the digestate.

This research thus aims to explore the potential of AD in the treatment of various organic wastes from diversified and intensified agricultural practices, particularly at low temperatures (20 ± 1 °C), and to promote sustainable waste management. It evaluates the effectiveness of a closed-loop, two-stage (solid-liquid) AD system for treating multi-substrate agricultural waste, improving its biogas production and digestate quality, while examining the impact of HM on organic matter before and after AD. The study proposes a unique method for routine monitoring of system performance and examines the effect of inoculum adaptation, offering comparisons with existing literature. This fills crucial knowledge gaps regarding low-temperature, multi-substrate AD systems suitable for cold regions and the effects of HMs on AD and the environment.

## Materials and methods

### Feedstocks and inoculum

In this study, different substrates were used: raw chicken manure (CM) with wood chip bedding and pig manure (PM) were obtained from a local farm in Quebec; raw dairy manure (DM) with straw bedding, from Agriculture and Agri-Food Canada (AAFC) in Sherbrooke; and wasted corn silage (CS), which was unable to feed the animals, were obtained from the AAFC farm. The inclusion of bedding materials aimed to provide a structural matrix beneficial for AD operations and to balance the C/N ratio (Babaee et al. 2013). CM and PM were rich in total ammonia nitrogen (TAN), while CS exhibited the highest soluble chemical oxygen demand (CODs) and the lowest pH, with minimal buffering capacity. The liquid inoculum (TS, 0.8%; VS, 0.3%, CODs, 2.7 g L^−1^; pH, 7.95; alkalinity, 9.5 g L^−1^ as CaCO_3_) was obtained from a laboratory-scale AD operation processing CM leachates. Table 1 summarizes the characteristics of the raw feedstocks and mixture used in all experiments.

**Table 1:** Physiochemical parameters of raw substrates used in all experiments

Cycle #	Raw feedstock and its mixture by set	TS, %	VS, %	TAN, g L^−1^	Organic N, g L^−1^	CODt, g L^−1^	CODs, g L^−1^	TVFA, g L^−1^	Alkalinity, g CaCO_3_ L^−1^	pH
1	Chicken manure	58.3	43.1	7.1	-	465.7	104.1	4.3	39.5	8.55
	Dairy cow manure	16.9	13.9	1.8	-	186.8	32.3	11.8	13.0	8.00
	Set 1 (CM)	51.6	38.7	6.9	-	738.1	92.5	3.4	35.2	8.6
	Set 2 (CM + DM)	26.5	19.5	4.8	-	297.0	50.0	6.1	25.8	7.6
2	Chicken manure	57.4	50.6	8.1	13.1	680.4	88.4	0.9	43.9	7.77
	Dairy cow manure	17.0	13.8	1.6	4.1	211.5	29.0	6.9	11.1	7.66
	Set 1 (CM)	28.3 ± 0.2	23.5 ± 0.2	5.5 ± 0.2	6.4	295.9 ± 10.7	37.5 ± 1.9	2.7 ± 0.2	27.5 ± 0.5	8.6 ± 0.1
	Set 2 (CM + DM)	22.8 ± 2.3	20.0 ± 2.2	3.5 ± 0.01	4.1	222.3 ± 35.8	26.9 ± 1.4	3.0 ± 0.1	18.4 ± 0.1	8.4 ± 0.1
3	Chicken manure	70.7	61.2	5.8	17.3	574.7	100.9	1.9	31.2	8.72
	Dairy cow manure	20.1	18.4	0.8	3.3	296.9	24.5	5.6	6.9	7.45
	Pig manure	9.4	7.5	5.1	1.5	80.4	31.5	12.1	19.0	7.28
	Corn silage	57.2	54.8	1.0	6.0	590.2	115.2	5.7	0	4.01
	Set 1 (M1)	28.8 ± 0.2	25.5 ± 0.2	2.9 ± 0.1	5.5	357.4 ± 21.2	37.9 ± 1.2	6.2 ± 0.3	14.4 ± 0.4	7.3 ± 0.0
	Set 2 (M2)	25.2 ± 0.6	22.1 ± 0.5	3.1 ± 0.0	4.8	324.7 ± 11.1	32.7 ± 0.6	6.7 ± 0.3	14.8 ± 0.2	7.0 ± 0.0

For all the raw substrates and during Cycle 1, the replicates were taken out from the same agglomerate; hence, values are not reported as avg. ± std. dev

### Experimental setup

**Fig. 1:**
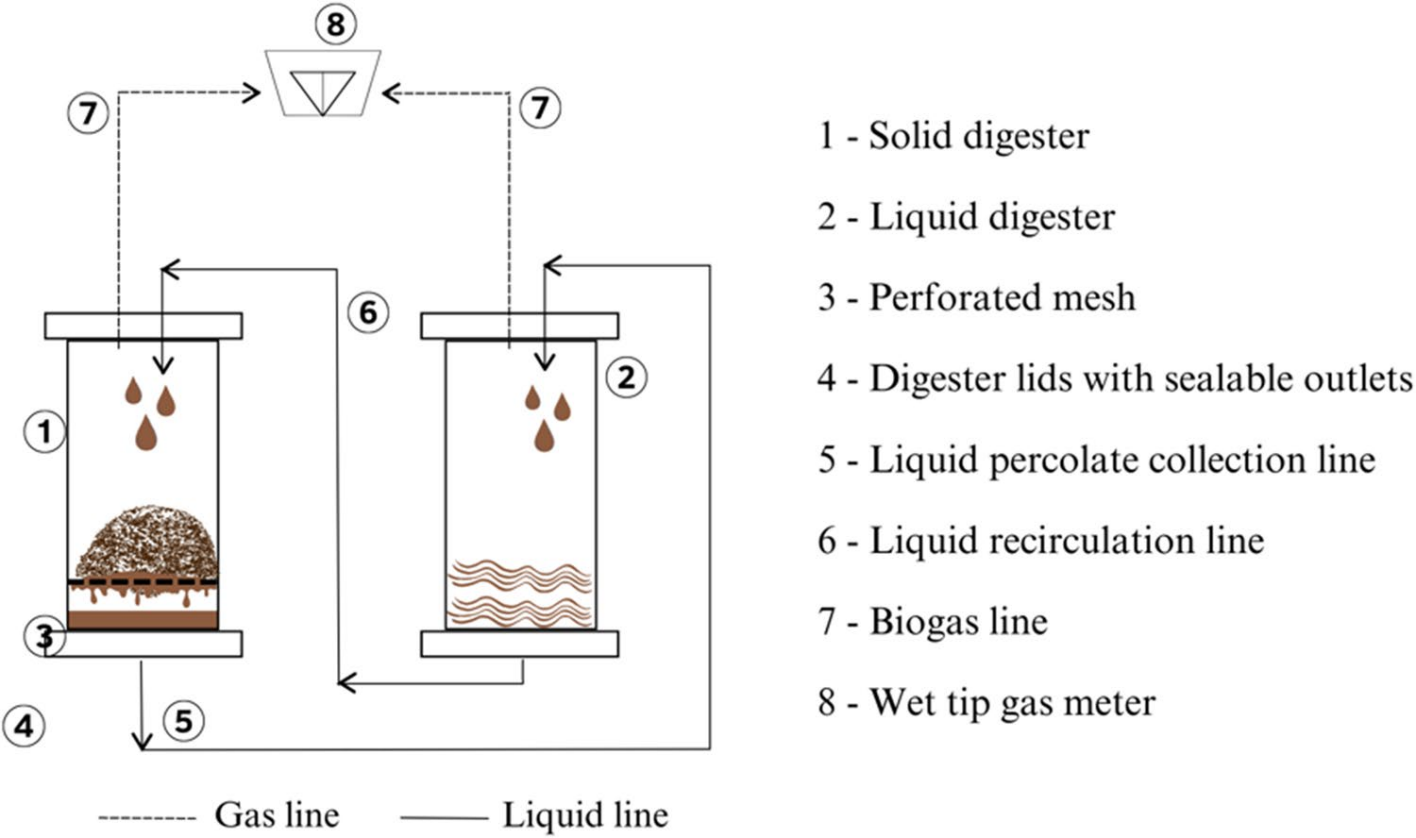
Two-phase (solid-liquid) anaerobic digester setup with percolation-recirculation mode of operation

**Fig. 2:**
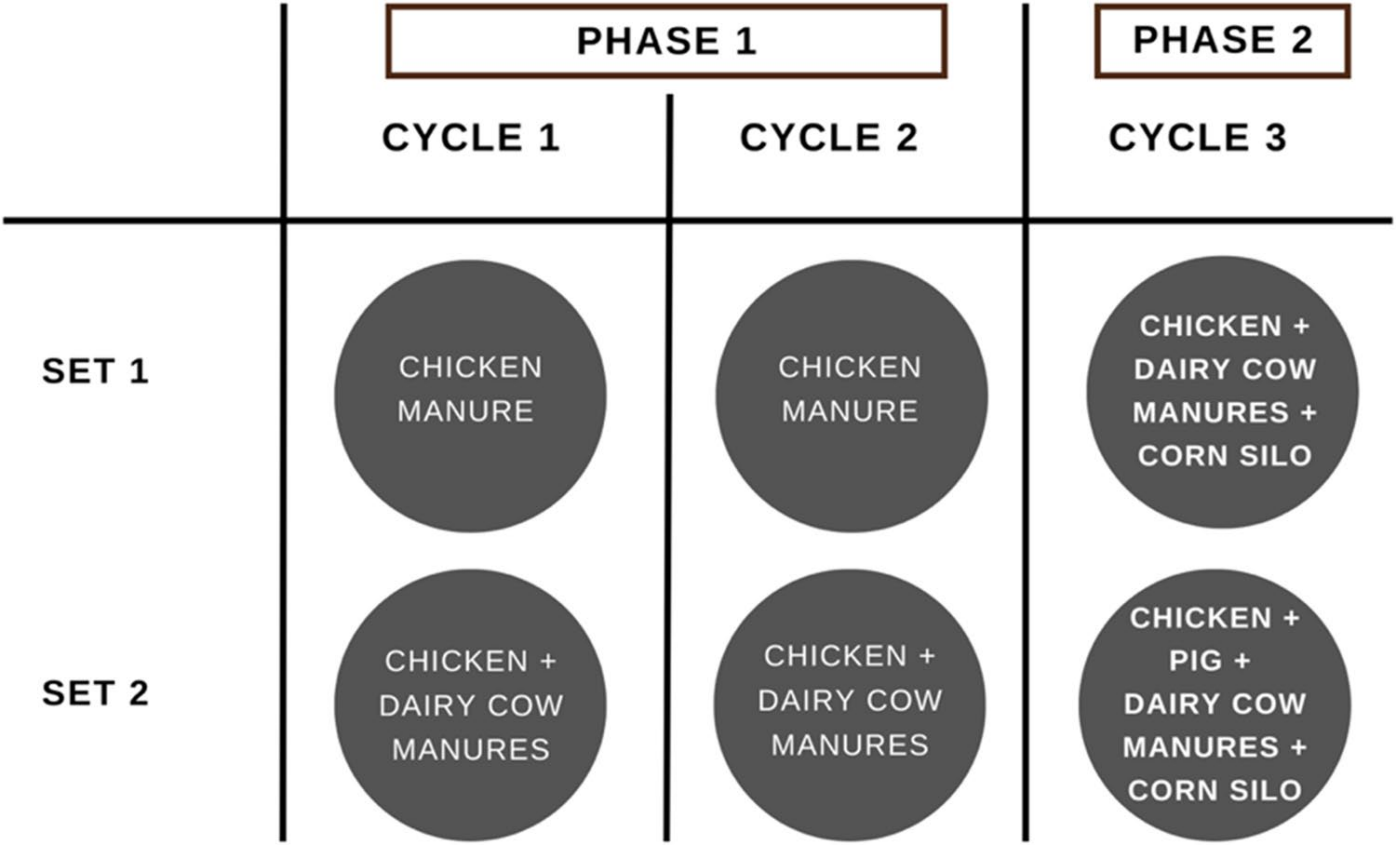
Graphical representation of the farm waste(s) treated in Sets 1 and 2 during all phases of this study

This study consisted of two phases involving different substrates. Phase 1 focused on the development of solid and liquid inoculums adapted to high nitrogen concentrations by mono-digestion of raw CM and co-digestion of CM with raw DM. Phase 2 involved multi-substrate AD, as described in Table 1. All experiments were carried out in duplicate using digester sets, each comprising a liquid digester and a solid digester interconnected for liquid recirculation and percolation mode of operation. Figure 1 illustrates the operation of the closed-loop, two-stage (solid-liquid) digester setup, whereas Fig. 2 demonstrates the operational phases according to the different manure mixtures used in this study, along with the specifics of cycles and sets. As shown in Fig. 2, Set 1 consisted of mono-digestion of CM in Cycles 1 and 2 and a multi-substrate digester in Cycle 3, whereas Set 2 consisted of co-digestion of CM + DM in Cycles 1 and 2 and a different multi-substrate digester in Cycle 3. Sets 1 and 2 were operated in parallel during each cycle. Phase 1 covered Cycles 1 and 2 (mono- and co-digestions), whereas Phase 2 covered the multi-substrate digestion under Cycle 3.

During the initial cycle (100 days), liquid inoculum derived from raw CM leachate in the laboratory was employed for mono- and co-digestions. The aim was to acclimatize the micro-organisms to high ammonia loads and high-solids content residues, and thus improve treatment efficiency. The solid and liquid digestates from Cycle 1 were then used to inoculate Cycle 2 (77 days), marking the end of Phase 1. During Phase 2 (Cycle 3, 68 days), the digestion of various substrates in different proportions was evaluated. Multi-substrates M1 and M2, comprising 50% solid inoculum from Cycle 2, raw DM, CM, CS, and PM (in M2) in varying mixing proportions, were used. Prior to digestion, the solid matrix was soaked overnight in liquid leachate or inoculum. Twice a week, a liquid inoculum of around 20–25% was circulated over the solid digester, with the percolated liquid recirculated into the liquid digester. The feed to inoculum ratio was between 1:5 and 1:5.5, maintaining a low operating temperature of 20 ± 1 °C. Table 2 shows detailed operating conditions, including feedstock proportions, organic loading rate (OLR), C/N ratio, and treatment period for both sets over three operating cycles.

**Table 2:** Operating conditions for all experiments in this study

Operating conditions/parameters	Phase 1				Phase 2	
	Cycle 1		Cycle 2		Cycle 3	
Substrate digestion strategy	Mono-digestion [Set 1]	Co-digestion [Set 2]	Mono-digestion [Set 1]	Co-digestion [Set 2]	Multiple substrates digestion [Set 1 (M1)]	Multiple substrates digestion [Set 2 (M2)]
Substrates used	CM	CM + DM	CM	CM + DM	CM + DM + CS	CM + DM + PM + CS
Cycle length (same as sludge retention time), days	100	100	77	77	68	68
Total volume of digester, L	40					
Raw material proportion (% w/w)	100	50:50	50	25:25	20:20:10	15:15:10:10
Total raw feedstock treated, kg	5	5	5	5	5	5
Raw manure treated, kg	5	5	2.5	2.5	2.5	2.5
Solid inoculum from the previous cycle, kg	-	-	2.5	2.5	2.5	2.5
Liquid inoculum, L	25	25	27.5	27.5	27.5	27.5
Substrate to Inoculum ratio	1:5	1:5	1:5.5	1:5.5	1:5.5	1:5.5
C/N (as COD_t_/TKN)	-	-	25.7	32.5	44.5	42.0
C/N (as COD_s_/TAN)	13.4	10.3	6.6	7.9	13.0	10.5
TVFA _initial feedstock,_ g L^−1^	3.4 ± 0.0	6.1 ± 0.0	2.7 ± 0.2	3.0 ± 0.1	6.1 ± 0.3	6.7 ± 0.3
OLR*, g VS L^−1^ d^−1^	4.3	2.8	4.5	3.1	5.1	4.6
Temperature, °C	20 ± 1					
Operation mode	Fed-Batch					

*OLR* organic loading rate; *conversion of mass to volume was done assuming the density of manure equal to the density of water (1 kg/L or 1 g/cc); values are based on raw substrate VS %, hence not reported as avg. ± std. dev

### Sampling and analysis

Samples from the solid digester (containing the substrate) were collected at the beginning and end of each cycle, while liquid digester samples were taken weekly to monitor the overall performance of the two-stage AD process. Biogas samples from both were collected thrice a week. Samples were analyzed for pH, alkalinity, TS, volatile solids (VS), total chemical oxygen demand (COD_t_), COD_s_, TKN (total Kjeldahl nitrogen), TAN, and VFAs. pH was measured using Mettler Toledo AG 8603 SevenMulti (Switzerland). Alkalinity was measured using Hach Lagne S Titralab AT1000 Series (Switzerland). COD was determined via closed reflux colorimetry. TKN and TAN were analyzed using the 2460 Kjeltec Auto-Sampler System (FOSS, Sweden). VFAs were determined using Perkin Elmer Clarus 580 (Perkin Elmer, Shelton, CT, USA), mounted with a DB-FFAP high-resolution column. The samples were conditioned according to the procedures mentioned by (Massé 2003) before evaluating the VFAs.

Basic statistical analysis (one-way ANOVA) was carried out using the Microsoft Excel Data Analysis Tool Pack. HMs like phosphorous (P), potassium (K), iron (Fe), and calcium (Ca) in raw and treated feedstock were analyzed at Université de Sherbrooke by Inductively Coupled Plasma Optical Emission Spectroscopy (ICP-OES) (PerkinElmer Avio 500). Biogas volume was recorded using wet-tip tank gas meter measurements. Biogas concentrations were analyzed using a gas chromatography (Micro GC 490, Agilent Technologies, Santa Clara, CA, USA) with a thermal conductivity detector. Helium was used as the carrier gas (20 mL/min), with injector and oven temperatures set at 110 °C and 180 °C, respectively.

Methane (CH_4_) calculations are taken into account once the methane concentration in the biogas reaches at least 40%. The specific methane yield (SMY, expressed as L CH_4_ g^−1^ VS_fed_ or L CH_4_ g^−1^ CODt_fed_) was calculated as the sum of the methane volumes produced by the solid and liquid digesters divided by the mass of raw organic matter (represented by VS_fed_ or CODt_fed_) in the digester, using Eqs. (1) and (2), as shown below. FAN was calculated using TAN, pH, and temperature from Eq. (3) as previously described by Hansen et al. (1998) and Mahato et al. (2020). The reduction of VFA and CODs was calculated using the formula given in Eq. (4).

1 $$SMY = \frac{ {\sum V}_{m}}{M}$$

where *V*_*m*_ is the sum of methane volumes produced from the solid and liquid digesters (L), and *M* is the mass of raw solid substrate fed, represented by g VS_fed_ or g CODt_fed_.

2 $$Methane \ volume\ produced \left({V}_{m}\right)= \sum {V}_{b} \times a$$

where *V*_*b*_ is the cumulative biogas produced by the liquid or solid digester (L), and *a* is the methane concentration in the biogas of the liquid or solid digester (%).

3 $$FAN=TAN {\left(1+ \frac{{10}^{-pH}}{{10}^{-\left(0.09018+\frac{2929.92}{T (K)}\right)}}\right)}^{-1}$$

where the *T* is the operating temperature in Kelvin (K); TAN and FAN are in (mg/L).

4 $$Reduction\ in\ VFA \ and\ CODs \left(\%\right)=\left(\frac{{X}_{1}-{X}_{2}}{{X}_{1}}\right) \times 100$$

*where X*_*1*_* and X*_*2*_* are the respective experimental initial and final values of CODs and total VFA.*

## Results and discussion

### Assessing digester efficiency: biogas and methane generation and yield

**Fig. 3:**
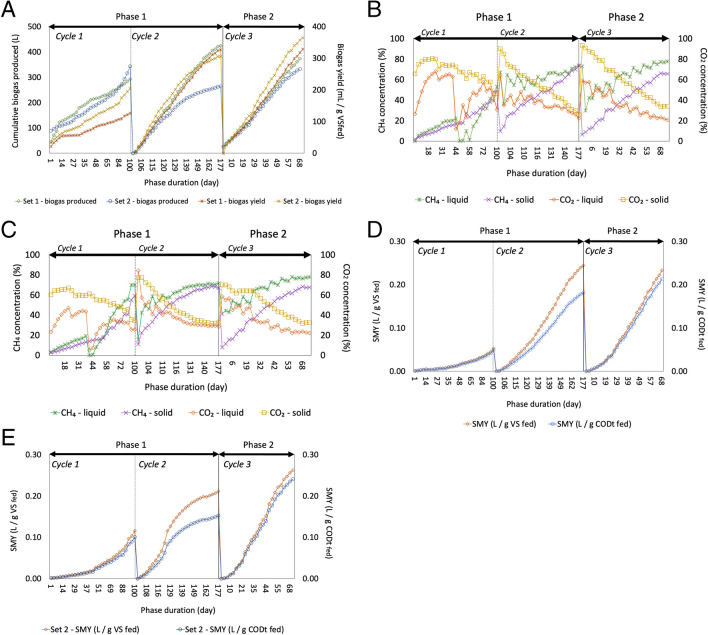
**A**–**E** Cumulative biogas production and biogas yield from both Set 1 and Set 2 (**A**). Methane and carbon-di-oxide concentration in biogas from liquid and solid digesters for Set 1 (**B**) and Set 2 (**C**). Specific methane yield (SMY) during both phases of this study from Set 1 (**D**) and Set 2 (**E**)

The efficiency of the digesters in terms of biogas and methane generation, as well as yield, was evaluated in this study. The performance of both solid and liquid digesters was analyzed across multiple operational cycles. Figure 3 provides a comprehensive overview of the cumulative biogas production, its yield, compositions (including methane and carbon dioxide concentrations), and SMY from each cycle for both types of digesters.

#### Overall performance of two-stage (solid + liquid) digesters

Despite high biogas production in Cycle 1, CH_4_ concentration was low while carbon dioxide (CO_2_) was relatively high. In particular, the first 100 days of operation (Cycle 1) were extended to facilitate inoculum adaptation to the multi-substrate mixture. This extension led to a significant increase in CH_4_ concentration to around 50% at the end of the cycle. Subsequently, once the CH_4_ concentration reached this level, the duration of the second cycle of operation was reduced to 77 days, leading to improved biogas and methane yields, as evident from Fig. 3A–E. The peak cumulative biogas production of 424.5 ± 4.0 L was achieved during Cycle 2 in Set 1, as illustrated in Fig. 3A. This facilitated the handling of the multi-substrate farm residue mixture in the third cycle of operation with an even shorter duration (68 days) without compromising the biogas and methane values, which can be attributed to the efficacy of the inoculum acclimation during Cycles 1 and 2.

The biogas yield during Cycle 1 was low, as anticipated; however, it improved over the next two cycles (Fig. 3A). By the end of Cycle 2, biogas yields of 327 mL g^−1^ VS_fed_ and 306 mL g^−1^ VS_fed_ from Sets 1 and 2, respectively, were reached, and by the end of Cycle 3, a biogas yield of 329 mL g^−1^ VS_fed_ and 365 mL g^−1^ VS_fed_ was obtained from Sets 1 and 2, respectively. In a continuous, mesophilic co-digestion study of four substrates, chicken litter (CL), food waste (FW), wheat straw (WS), and hay grass (HG), the highest biogas yield (normalized for standard temperature and pressure, STP) was attained at an OLR 2.0 g TS L^−1^ d^−1^ from the combinations: (CL+FW+WS) 350.5 ± 10.1 mL_N_ biogas g^−1^ VS_fed_, (CL+FW+HG) 300.2 ± 13.1 mL_N biogas_ g^−1^ VS_fed_, and (CL+FW+WS+HG) 290.7 ± 9.3 mL_N_ biogas g^−1^ VS_fed_ (Zahan et al. 2018). These results are in line with those obtained for multiple substrate digestion in this study.

#### Performance evaluation of liquid digesters

Concerning the liquid digesters exclusively, Fig. 3B and C exhibit a notable trend of higher CH_4_ and CO_2_ concentrations in the biogas compared to their solid counterparts. Throughout the AD process, the solids being treated displayed a gradual descent in CO_2_ levels and a corresponding ascend in CH_4_ concentration, a pattern less pronounced in the liquid digesters. A plausible explanation for this phenomenon is the increased mobility and nutrient exchange of the liquid phase due to recirculation and percolation over the solid matrix. In Cycle 1, Set 2 has slightly higher (approx. 10%) methane levels perhaps due to a positive effect of co-digestion. In Cycle 2, Set 1 exhibited a marginal increase (approx. 6%) in methane concentration in biogas compared to Set 2. This difference is attributed to the well-adapted liquid inoculum (in case of high-N CM) in Cycle 2, resulting in a higher biogas production and SMY compared to Set 2. By the end of Cycle 3, the methane concentration from Sets 1 and 2 was similar despite the variation in the composition of the substrates. In yet another mesophilic co-digestion study of 60 days, organic waste produced locally on an Island in Portugal, namely, cow manure, pig slurry, bird manure, kitchen waste, sewage sludge, oily lacteous waste, was used (Fernández-Rodríguez et al. 2023). Methane concentrations ranged from 68.7 to 73.3%, with up to six different wastes (or substrates) used, although no more than three substrate mixtures were co-digested together.

The methane and biogas volume, along with the SMY, produced for all digesters during the three operational cycles are summarized in Table 3. Methane calculation began when its concentration in the biogas reached a minimum of 40% during each cycle. SMY was lowest in Cycle 1, for Sets 1 and 2, with its value of 0.048 ± 0.004 and 0.100 ± 0.018 L g^−1^ COD_Tfed_, respectively, as shown in Fig. 3D and E. Nevertheless, a marginal benefit of co-digestion over mono-digestion was observed in terms of a higher SMY and average methane concentration in biogas during Cycle 1. In Cycle 2, however, Set 1 had higher SMY than Set 2, highlighting the efficacy of the inoculum used, particularly for high-N CM, and corroborated with the reduction in treatment length seen and stable AD process despite an increased OLR. In Cycle 3, not much difference was observed in the SMY for Sets 1 and 2 which were 0.233 ± 0.028 L g^−1^ VS_fed_ and 0.262 ± 0.004 L g^−1^ VS_fed,_ respectively. A study conducted for 65 days on mesophilic co-digestion of pig manure, corn stover, and cucumber residues, in a 5:2:3 proportion, gave the highest methane yield of 305.4 mL g^−1^ VS (Wang et al. 2018); these results are along similar lines with the findings from the present study under Phase 2, i.e., for multi-substrate AD. The differences in the methane yield occur due to the different physiochemical parameters of the mix of substrates and different operating conditions.

**Table 3:** Biogas production and specific methane yield for all digesters over three operating cycles

Cycle #	Set #	Combined* cumulative biogas production, L	Total methane generated, L		Average CH_4_ concentration in biogas, %		Combined* SMY, L g^−1^ VS_fed_	Combined* SMY, L g^−1^ CODt_fed_
			Liquid	Solid	Liquid	Solid		
1	1	343.8 ± 8.4	48.2 ± 0.9	44.3 ± 0.5	35.4 ± 18.4	22.7 ± 13.7	0.018 ± 0.005	0.048 ± 0.004
	2	307.8 ± 8.5	61.3 ± 1.2	41.8 ± 0.5	41.7 ± 22.2	28.2 ± 18.8	0.116 ± 0.021	0.100 ± 0.018
2	1	424.5 ± 4.0	102.1 ± 1.3	127.4 ± 1.9	64.0 ± 12.2	50.0 ± 19.0	0.244 ± 0.011	0.181 ± 0.008
	2	264.0 ± 3.9	48.5 ± 1.0	90.9 ± 1.3	61.6 ± 13.6	53.8 ± 16.8	0.211 ± 0.019	0.152 ± 0.013
3	1	373.4 ± 5.8	124.9 ± 2.6	60.0 ± 1.7	59.1 ± 17.5	36.6 ± 20.2	0.233 ± 0.028	0.214 ± 0.026
	2	332.0 ± 5.2	108.3 ± 2.3	61.3 ± 1.3	60.5 ± 17.5	40.3 ± 21.0	0.262 ± 0.004	0.241 ± 0.004

Calculations concerning methane began when the methane concentration in biogas had reached a minimum of 40%

^*^Combined SMY is the sum of individual-specific methane yields from liquid and solid digesters

Thus, the adaptation of the inoculum significantly enhanced the efficiency of two-stage AD in this study. Particularly noteworthy was the superior performance of the adapted inoculum in Cycle 2, Set 1, which contributed to increased biogas production and SMY, aligning with findings by Lendormi et al. (2022) and Mahdy et al. (2017). This highlights the importance of tailored inoculum selection for optimizing methane production, especially at lower temperatures. Moreover, the adapted inoculum demonstrated versatility in digesting multiple substrates, as evidenced in Cycle 3, which aligns with the findings of Zahan et al. (2018). These results underscore the potential of specific inoculum development for multi-waste streams, facilitating continuous operation and advancing sustainable waste management and renewable energy production.

### Short-chain fatty acid evolution

Table 4 presents the short-chain fatty acids (C2, acetic acid; C3, propionic acid; iC4, isobutyric acid; C4, butyric acid; iC5, isovaleric acid; and C6, valeric acid), along with their initial and final total VFA concentrations for both the liquid and solid digesters of Sets 1 and 2. During Cycle 1, overall performance was poor, with VFA accumulation observed toward the end. This result was expected due to the absence of acclimatized solid inoculum and high levels of total ammonia nitrogen (6.91 g L^−1^ and 4.83 g L^−1^ in sets 1 and 2, respectively). Furthermore, recirculation-percolation of the liquid inoculum through the solid matrix contributed to higher VFA levels in the liquid digesters. In Set 1, the final VFA concentration in the solid digester reached a maximum of 14.0 g L^−1^ from an initial value of 3.4 g L^−1^, while VFA decreased marginally in Set 2. In Cycle 2, VFA consumption and COD reduction in the feedstock varied from 72% to 73% and 77%, respectively. Both sets of digesters showed no signs of inhibition due to the accumulation of short-chain fatty acids during this cycle.

**Table 4:** Initial and final VFA concentrations in the liquid and solid digesters of Cycle 3

Set/measurement specifications		TVFA	Acetic (C2)	Propionic (C3)	Isobutyric (iC4)	Butyric (C4)	Isovaleric (iC5)	Valeric (C5)	Caproic (C6)
*Liquid digesters*									
Set 1 (M1)	Initial	93.9 ± 12.2	70.7 ± 13.1	5.6 ± 0.9	0.0	8.4 ± 2.5	0.0	0.0	9.1 ± 0.6
	Final	695.2 ± 101.9	495.3 ± 83.0	139.2 ± 98.1	3.2 ± 2.3	1.7 ± 1.2	9.5 ± 7.5	0.0	9.3 ± 6.6
Set 2 (M2)	Initial	71.5 ± 8.5	61.5 ± 3.0	0.0	0.0	5.5 ± 0.9	0.0	0.0	0.0
	Final	71.5 ± 1.3	57.5 ± 25.1	3.5 ± 2.5	0.0	5.1 ± 1.5	0.0	0.0	8.5 ± 0.5
*Solid digesters*									
Set 1 (M1)	Initial	6179.1 ± 291	5452.4 ± 48.5	271.3 ± 22.2	73.9 ± 26.2	96.6 ± 35.6	80.9 ± 32.8	0.0	154.4 ± 55.4
	Final	3933.6 ± 757.9	1945.4 ± 476.7	1026.9 ± 435.7	169.1 ± 20.1	142.2 ± 36.9	300.2 ± 18.4	140.9 ± 40.2	208.7 ± 38.9
Set 2 (M2)	Initial	6672.7 ± 291.8	5627.0 ± 376.8	395.9 ± 24.3	114.9 ± 6.3	205.5 ± 30.7	151.4 ± 6.9	0.0	143.5 ± 16.7
	Final	2448.2 ± 770.5	1524.4 ± 302.4	525.1 ± 443.6	65.1 ± 8.4	80.4 ± 2.2	140.4 ± 40.2	0.0	112.9 ± 21.8

^*^All units in mg L^−1^

As far as Phase 2 (Cycle 3) is concerned, the VFA evolution proceeded well in the multi-substrate digesters. A prolonged treatment period can facilitate greater VFA consumption in the Set 1 digesters, with no signs of inhibition observed during this phase. While both sets of digesters demonstrated efficiency, variations in their initial and final VFA levels were noticeable. It should be noted that the performance of the entire two-stage AD system was effectively monitored by regular sampling of the liquid digester. Thanks to the recirculation-percolation mode of operation once or twice a week, it was possible to monitor the efficiency of the solid digesters via the liquid digesters. In a healthy anaerobic system, the VFA concentration in the effluent should be low (50–250 mg Hac L^−1^). More importantly, the unionized form of VFAs (like acetic, propionic, and butyric acids) can inhibit methanogens when present at concentrations of 30–60 mg L^−1^ (Khanal 2008). The final VFA concentration of the Set 1 liquid digester can be compared to its day 0 value to see the rise or decline. In this case, a 4.6-fold increase in the total VFA value was observed, with a concentration of 495.3 ± 83.0 mg L^−1^ as HAc. This result indicates pending VFA consumption in the solid digester, which was reflected after observing the final VFA levels in the solid digester at the end of the treatment period. The final VFA concentration of the Set 2 liquid digester was lower than its day 0 value and suggested better VFA consumption in its solid digester.

Based on the above information, a hypothesis can be postulated for the impending VFA consumption in the solid digester. With regard to individual fatty acid concentrations at the beginning and end of the treatment period for solid digesters in Cycle 3, Set 2 showed a decrease in all short-chain fatty acid concentrations towards the end of their treatment period compared with their initial values (Table 4). However, Set 1 revealed an increase in all the short-chain fatty acid concentrations toward the end of their treatment period, except in acetic acid concentration, suggesting a rapid adaptation of methanogens. Meanwhile, the breakdown of intermediary products (C3–C6) to acetate (C2) was slower, suggesting a slow adaptation of acetogens. This hypothesis is supported by the low total VFA consumption (≈43%) in the M1 solid digester (see Table 4), which indicates the impending VFA consumption in Set 1. Therefore, a slightly longer treatment duration is suggested.

### Performance monitoring using ratio limits

**Fig. 4:**
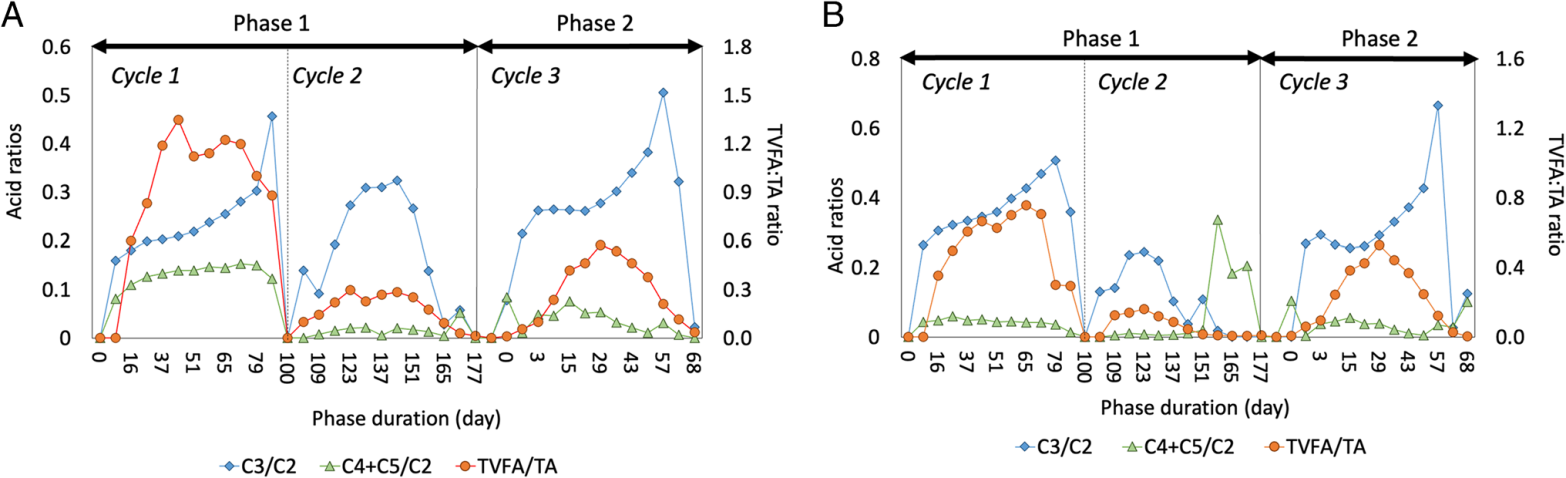
**A**, **B** Stability ratios for Set 1 (**A**) liquid digesters and Set 2 (**B**) liquid digesters

A TVFA/alkalinity ratio less than 1 indicates stable AD, while 0.4–0.6 is considered optimal (Mahato et al. 2020, 2022). A ratio of 0.8 and above lowers pH and hinders methanogenesis (Khanal 2008). Low TVFA/alkalinity ratios ensure sufficient buffering capacity. A digester failure can occur at a propionic-to-acetic acid ratio (C3/C2) greater than 1.4 or at the ratio of the sum of butyric and valeric acids -to-acetic acid ratio [(C4+C5)/C2] above 0.3 (Hill and Holmberg 1988; Nuchdang and Phalakornkule 2012; Mahato et al. 2020, 2022). Ratios for all experiments are in Fig. 4A and B. For Set 1 digesters during Cycle 1 (mono-digestion, CM), the TVFA/alkalinity ratio approached 1.4, indicating instability due to lack of active microorganisms, requiring longer digestion (100 days). High N levels in feedstock likely shocked the digester. However, other experiments maintained optimal ratios. C3/C2 remained below 1.4 for all phases. (C4+C5)/C2 below 0.3 suggests stable AD. Set 2 digester (co-digestion of CM+DM) during Cycle 2 reached (C4+C5)/C2 ≈ 0.34, with no hindrance in digestion despite butyric acid accumulation. Microbial analysis revealed different bacteria involved in C3 and C4 oxidation to C2 (Lim et al. 2020). Possibly there was a lower number of butyrate-degrading bacteria, which could be the reason for its accumulation, future studies could further explore this.

### Relationship between chemical parameters of liquid digesters from Phases 1 and 2

**Fig. 5:**
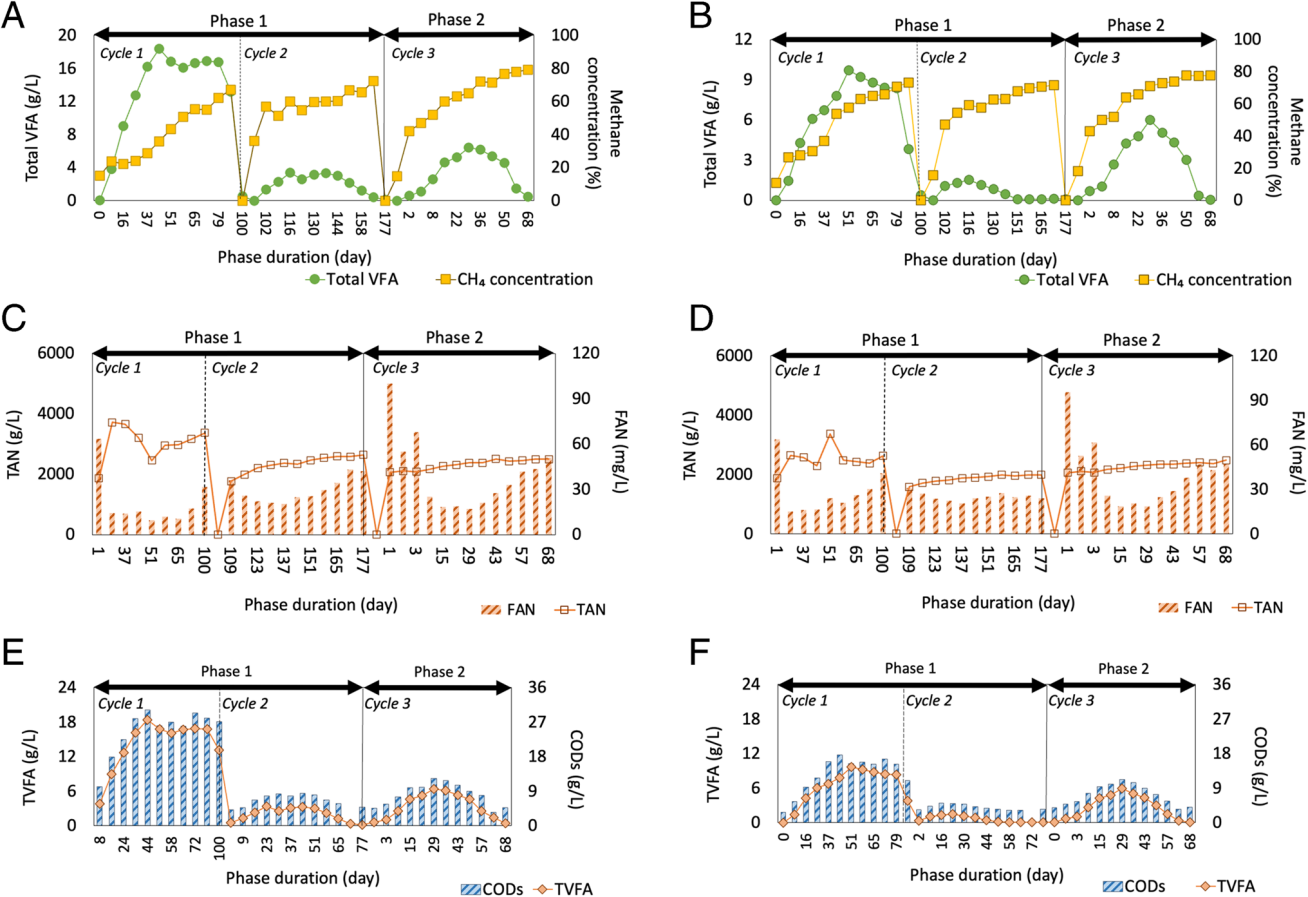
**A**–**F** Total VFAs consumption vs methane concentration from the liquid digesters of Set 1 (**A**) and Set 2 (**B**). Free and total ammonia nitrogen concentrations in the liquid digesters of Set 1 (**C**) and Set 2 (**D**). TVFA and CODs during each cycle for the liquid digesters of Set 1 (**E**) and Set 2 (**F**)

The relationship between the chemical parameters of liquid digesters in Phases 1 and 2 reveals significant insights. Alkalinity increases during anaerobic decomposition of organic nitrogen, with a reduction in organic nitrogen concentration in the feedstock post-treatment. Phase 2 consistently displayed this trend, whereas Phase 1, Cycle 1, experienced VFA accumulation and decreased alkalinity due to FAN inhibition. Although the organic N concentration in Cycle 1 feedstock was unavailable, signs of inhibition were evident, leading to decreased alkalinity. The evolution of pH and alkalinity across cycles is detailed in Supplementary information (Fig. S1). The relationship between various parameters like TAN, FAN, VFA, and CH4 concentration have been shown in Fig. 5A–F. The relationship between VFAs and methane concentration demonstrates increasing VFA consumption over cycles, while methane concentration remains consistent, see Fig. 5A and B. Cycle 3 exhibited slightly higher VFAs attributed to the addition of corn silo and pig manure. Moreover, solid digesters in Set 1 had lower pH and total VFA concentration but higher organic N and COD levels compared to Set 2, reflecting differences in raw feedstock composition. Figure 5C and D depict TAN and FAN concentrations in liquid digesters across cycles, mostly below threshold values.

Phase 2’s stability prompted a rudimentary statistical analysis, revealing a negative correlation between FAN concentration and average daily biogas production rate, consistent with prior studies. Additionally, FAN and total VFA displayed a strong negative correlation, while TVFA exhibited a positive correlation with the average daily biogas production rate. A strong positive correlation was observed between TVFA and COD concentrations, suggesting an increase in soluble organic matter with VFA breakdown. Figure 5E and F illustrate VFA and COD levels during each cycle for Sets 1 and 2, indicating consumption of readily available organic matter followed by complex compound breakdown. COD removal rates were similar across Sets 1 and 2, while VFA consumption was lower in Set 1, possibly due to slightly higher organic N content.

This comprehensive analysis sheds light on the intricate relationships between various chemical parameters in liquid digesters across different phases and cycles of the experiment. Understanding these relationships is crucial for optimizing anaerobic digestion processes and improving biogas production efficiency.

### Heavy metal significance and implications

Several research works have demonstrated the positive effect of HMs at low concentration and their effect on AD. Guo et al. (2019) found that low concentrations of Cu^2+^ (0–100 mg L^−1^), nickel (Ni^2+^) (0.8–50 mg L^−1^), Fe^2+^ (50–4000 mg L^−1^), cadmium (Cd^2+^) (0.1–0.3 mg L^−1^), and Zn^2+^ (0–5 mg kg^−1^) promote microbial activity during AD increasing the biogas production, while high concentrations inhibit AD process (Zhang et al. 2016; Zhang et al. 2018). The ICP-OES results indicate the HMs concentration change during each cycle and are summarized in Table 5. HM concentrations fluctuated across cycles, decreasing at the end of Cycles 1 and 3 but increasing in Cycle 2, except for K. This trend aligns with findings from previous studies (Selling et al. 2008; Golovko et al. 2022; Kadam et al. 2022) highlighting HM enrichment in digestates. While HM concentrations remained within permissible limits for biofertilizer use, long-term application could pose environmental risks. Studies on mesophilic AD of PM and waste-activated sludge also reported higher HM concentrations in digestates compared to raw substrates (Kadam et al. 2022; Shamsollahi et al. 2019).

**Table 5:** Heavy metal concentrations in the raw feedstock and digestate

Cycle #	Baseline measurement details	Feedstock and set details	Heavy metals						
			Al 394.401 (mg/L)	Cu	K	Mg	Mn	P	Zn
1	Initial value	Raw CM	0.84	1.75	24.35	13.54	1.16	35.16	1.00
	Initial value	Raw DM	0.45	0.07	10.96	4.46	0.05	6.68	0.07
	End of treatment	Set 1	0.61 ± 0.1	0.97 ± 0.2	6.42 ± 0.9	6.08 ± 1.2	0.43 ± 0.1	15.91 ± 2.7	0.47 ± 0.1
	End of treatment	Set 2	0.58± 0.1	0.64 ± 0.1	5.49 ± 0.9	5.65 ± 1.6	0.35 ± 0.1	14.19 ± 4.2	0.32 ± .01
2	Initial value	Raw CM	0.42	0.77	12.31	5.51	0.44	16.56	0.37
	Initial value	Raw DM	0.19	0.08	7.06	3.97	0.07	6.39	0.11
	End of treatment	Set 1	0.77 ± 0.01	0.97 ± 0.01	6.38 ± 0.9	9.51 ± 1.5	0.69 ± 0.1	23.53 ± 0.7	0.61 ± 0.2
	End of treatment	Set 2	0.91 ± 0.4	0.78 ± 0.2	5.98 ± 1.4	11.34 ± 3.7	0.61 ± 0.2	23.86 ± 5.0	0.60 ± 0.3
3	Initial value	Raw CM	1.00	1.40	23.58	12.06	0.76	29.03	0.61
	Initial value	Raw DM	0.16	0.04	5.71	3.75	0.04	4.73	0.06
	Initial value	Raw CS	0.13	0.06	8.88	3.47	0	8.02	0.02
	Initial value	Raw PM	0.82	0.95	0	0	1.02	95.14	2.36
	End of treatment	Set 1	0.69 ± 0.1	0.63 ± 0.1	5.41 ± 0.8	5.59 ± 0.5	0.31 ± 0.01	12.42 ± 1.8	0.31 ± 0.01
	End of treatment	Set 2	0.49 ± 0.01	0.53 ± 0.0	4.83 ± 0.6	5.58 ± 0.1	0.29 ± 0.01	13.14 ± 0.6	0.29 ± 0.01

^*^All units in mg L^−1^ and the values shown in Set 1 and Set 2 are the averages of its two replicates

In Cycle 3, PM exhibited higher concentrations of Zn and P, while CM had higher levels of Ca, Cu, K, magnesium (Mg), manganese (Mn), and P. Negligible amounts of Cd, chromium (Cr), cobalt (Co), and Ni were detected. Despite the initial high concentrations in PM, both P and Zn decreased significantly by the cycle’s end. Set 1 showed slightly higher HM concentrations compared to Set 2 (except for P). The lower VFA removal from solid feedstock in Set 1 (43%) suggested that some HMs remained unused by microorganisms, reducing microbial VFA consumption. Liquid recirculation likely facilitated HM mobilization to the liquid digester. Future studies should evaluate HMs in liquid samples, with the potential for valuable HM recovery from liquid digestate through adsorption (Selling et al. 2008).

The digestate obtained at the end of a treatment cycle could have an accumulation of HM which presents a potential obstacle to its use as a biofertilizer (Selling et al. 2008) because its application may circulate these elements back into the food chain and could have negative impacts on living organisms (Golovko et al. 2022); hence, the reduction in HMs is one of the essential parameters for its use as a biofertilizer (Zhu et al. 2014). As per the European Union directive for biofertilizers, the permissible values for cadmium (Cd), Cu, Ni, and Zn are 1, 100, 50, and 200 mg kg^−1^, respectively (Kadam et al. 2022). Nevertheless, the solid digestate could be used as a biofertilizer as the concentration of HM was within the permissible limits; however, it is one of the quality assessment factors when testing biofertilizer potential.

### Comparison with other low temperature and psychrophilic studies

Table 6 provides a comprehensive overview of studies conducted at temperatures ranging from 10 to 28 °C, encompassing various feedstocks such as manures, crop residue, swine carcass, and municipal wastewater, predominantly within reactors like plug-flow, membrane bioreactors, and batch reactors. Notably, research on multiple substrate digestion at low temperatures remains limited, underscoring the significance of this study in advancing sustainable livestock farming and bioenergy production. Successful treatment of high-ammonia wastes such as chicken manure (CM) and swine manure at 20 °C has been demonstrated through the utilization of an adapted inoculum and the mode of start-up operation, resulting in SMY of 0.52 ± 0.13 L g^−1^ VS_fed_ and 0.49 ± 0.10 L g^−1^ VS_fed_, respectively (Massé et al. 2014; Mahato et al. 2020).

**Table 6:** Operating conditions and specific methane yield of anaerobic digesters at low or ambient temperature

Feedstock	Reactor type	Cycle length, day	Temperature, °C	OLR, g VS L^−1^ d^−1^	SMY, L g^−1^ VS _fed_	Remarks	Reference
*Studies conducted at AAFC, Sherbrooke*							
Cow and chicken manure (co-digestion)	Batch	112, 78	20	3.7, 4.7	0.35 ± 0.11	• Cycles 1, 2 = 112, 78 days • TS 65%, and 73% for Cycles 1 and 2, respectively, (co-digestion) • TS 48%, and 51% for Cycles 1 and 2, respectively, (CM) • Co-digestion gave better methane-rich biogas than mono-digestion, but SMY from mono-digestion was higher	Mahato et al. (2020)
Chicken manure (CM)	Batch	112, 78	20	4.3, 4.6	0.52 ± 0.13		
Swine manure	Sequencing batch (SBR)	28	20	1.2	0.266 ± 0.014*	• The temperature was decreased from 20 to 15 to 10 °C (in the first three tests) then raised back to 15 °C and then to 20 °C (in the last two tests) • The operating temperature was changed gradually over 5 days • When the temperature was raised again, the methane production increased to 0.125 ± 0.007 (15 °C) and 0.214 ± 0.013 (20 °C) from 0.080 ± 0.002 (10 °C) • Temperature is directly proportional to SMY (SMY decreased significantly at 10 °C) but once the temperature was increased back to 20 °C, the system was able to recover from the temperature shock	Massé (2003)
Swine manure	SBR	28	15	1.2	0.218 ± 0.022*		
Swine manure	SBR	26 - 37	10	1.1	0.080 ± 0.002*		
Pig manure (PM) + NH_4_Cl	SBR	28	24.5 ± 0.5	3.0 ± 0.35^a^	0.48 ± 0.09	• AD process at high ammonia levels was successfully studied in the long-term • Total ammonia (as NH_3_-N) concentration: 8.2 ± 0.3 g L^−1^ for (PM + NH_4_Cl) reactor and 5.5 ± 0.7 g L^−1^ for control reactor • Average methane concentration in biogas: 68.3 ± 2.4% for (PM + NH_4_Cl) reactor and 70.2 ± 2.9% for control reactor • Longer solids/hydraulic retention times can enhance biomass acclimation even at high-ammonia levels	Massé et al. (2014)
Pig manure (control)	SBR	28	24.5 ± 0.5	3.0 ± 0.35^a^	0.49 ± 0.10		
Wheat straw (WS)	SBR	21	20	0.6–2.0^b^	0.129 ± 0.017$	• Single reactor system anaerobically treated high-solids with TS content in WS: 89%, in CF: 10–11%, in the mix: 16% • 3 cycles, 21 days each • The average methane concentration in the biogas was, WS: 40.3 ± 0.7%, CF: 47.7 ± 9.7%, and mix: 48.7 ± 10.0% • Hydrolysis was the rate-limiting step	Massé et al. (2015)
Cow feces (CF)	SBR	21	20	0.6–1.9^b^	0.164 ± 0.023$		
Cow feces + Straw (mix)	SBR	21	20	0.6–2.4^b^	0.152 ± 0.006$		
Ground swine carcasses and swine manure slurry	SBR	28	25	3.2^a^	0.334*#	• One 4-week cycle and two 2-week cycles were performed subsequently • Performance of all three reactors (co- and mono-digestion) was the same statistically in terms of biogas production and quality • Carcass material was successfully converted into methane-rich biogas and high COD reductions were also achieved	Massé et al. (2008)
Ground swine carcasses and swine manure slurry	SBR	28	20	3.2^a^	0.273*#		
Swine manure slurry	SBR	28	25	3.2^a^	0.288*#		
Swine manure	Plug-flow reactor (PFR)	371	25	2.4 ± 0.2^c^	0.397–0.482#$	• Theoretical HRT 67 ± 7 days • After 150 days of treatment, AD performance fluctuated due to the accumulation of solids in the upstream compartments of the PFR; this prevented the complete digestion in the first few compartments • The reactor design needs to be modified to better manage the accumulating solids	Massé et al. (2013)
Cow feces + Wheat straw	SBR	21	20	5.1^b^	0.147 ± 0.017#$	• TS of the mixture 27% • Four consecutive cycles (21 d each) • Inoculum-to-substrate ratio (VS-based) of 1.6 at OLR of 5.1 g_VSfed_ kg^−1^_inoculum_ d^−1^ and 1.4 at OLR of 5.8 g_VSfed_ kg^−1^_inoculum_ d^−1^ • Hydrolysis was the limiting step reaction	Massé and Saady (2015a)
Cow feces + Wheat straw	SBR	21	20	5.8^b^	0.143 ± 0.011#$		
Cow feces	SBR	21	20	5.9–7.9^b^	0.154 ± 0.011–0.116 ± 0.006#$	• TS 11.5–13.5% • Inoculum-to-substrate ratio (VS-based) ranged between 0.48 and 0.74 • Hydrolysis was the rate-limiting reaction • A longer treatment cycle length is required for higher OLR (> 7.0 g_VS_ kg^−1^_inoculum_ d^−1^)	Massé and Saady (2015b)
CM	Batch	100, 77	20 ± 1	4.3, 4.5	0.052, 0.244	• It took 100 days to adapt the inoculum to perform at 20 ± 1 °C • With an adapted inoculum, mixtures of multiple different wastes were successfully treated to produce biogas • Adapted inoculum helped to reduce the treatment time even when OLR was increased	This study
CM, DM	Batch	100, 77	20 ± 1	2.8, 3.1	0.076, 0.211		
CM, DM, CS	Batch	68	20 ± 1	5.1	0.232		
CM, DM, PM, CS	Batch	68	20 ± 1	4.6	0.262		
*Other studies*							
Swine slurry	Batch	9 months	13	n/a	0.125#%	• One mesophilic digestate and four manures (fresh and stored) were used as inoculum • After 9 months of acclimation, SMY reached only 125 N L_CH4_ kg^−1^_COD added_	Lendormi et al. (2022)
Cattle manure + Corn straw	Batch	≈66	20 ± 1	n/a	0.036$ Control (i.e., no bioaugmentation) 0.0l8$	• The highest methane yield obtained was 36 mL g^−1^_VS_ • 2 L bioreactor, working volume 800 mL • Propionate-degrading bioaugmentation culture was used that helped to avoid VFA accumulation (acetate and propionate especially) • The recommended dose for high biogas production was 14% (based on the VS ratio of bioaugmentation seed and feedstock, g VS_BS_ g^−1^ VS_FS_) and 4% for the highest bioaugmentation efficiency of microbes • In bioaugmentation reactors, an increase in the proportions of bacteria (propionate-oxidizing, syntrophic butyrate-oxidizing) and acetoclastic methanogens was observed	Xu et al. (2022)
Municipal wastewater	Submerged anaerobic MBR (SAMBR)	33, 35, 39	15, 20, 25	n/a	0.06 ± 0.01, 0.14 ± 0.03, 0.17 ± 0.01^	• 20 L SAMBR, HRT 6 h • Poor performance at 15 °C and HRT 6 h • Electric energy generated was 0.67 kWh d^−1^ (at 15 °C) and 1.82–2.27 kWh d^−1^ (at 20–25 °C) • Even during temperature changes, acetoclastic methanogenesis was the main pathway	Ji et al. (2021)
Dairy manure	Batch	35	20	n/a	0.368&	• Used biochar to improve AD kinetics • The addition of biochar shortened the lag phase of AD and lowered the concentrations of VFAs (especially propionic acid) • A higher dose of biochar (10 g L^−1^) gave a higher methane yield • Control, ≈290 mL g^−1^_VS removed_	Jang et al. (2018)

*CM* chicken manure, *DM* dairy cow manure, *PM* pig manure, *CS* corn silage

^*^L g^−1^_TCOD fed_

^#^Normalized value at STP

^$^Converted to L g^−1^_VSfed_

%L g^−1^_COD added_

^L g^−1^_COD removed_

^&^L g^−1^_VS removed_

^a^g _COD_ L^−1^ d^−1^

^b^g _VS_ kg^−1^_inoculum_ d^−1^

^c^g _TCOD_ L^−1^_reactor_ d^−1^

Studies by Lendormi et al. (2022) and Ji et al. (2021) further exemplify the challenges and opportunities in low-temperature AD. Lendormi et al. explored psychrophilic AD of swine slurry, achieving a maximum methane yield of 42 L CH4 kg^−1^ VSsubstrate. d^−1^ at 13 °C. Ji et al. utilized a submerged anaerobic (SA) membrane bioreactor (MBR) for treating municipal wastewater at temperatures ranging from 15 to 25°C, demonstrating effective organic removal but facing challenges at 15 °C.

Instances of inhibited steady-state conditions during AD of farm waste, denoting a stable yet suboptimal state due to inhibitory factors like low carbon-to-nitrogen (C/N) ratio, have been documented (Nie et al. 2022). This inhibition adversely affects methane production efficiency and overall organic matter degradation, underscoring the potential of co-digestion with multiple substrates to enhance the AD process by balancing the C/N ratio.

In summary, this comparative analysis highlights ongoing research efforts to optimize AD technology for sustainable waste management and renewable energy production. As the FAN, a hindrance in AD is influenced by pH and temperature; Mahato et al. (2020) found that increasing operating temperature released more FAN from high-nitrogen wastes such as CM. Massé et al. (2014) spiked swine manure with NH_4_Cl, showing no VFA accumulation due to pre-acclimatized inoculum use. In this study, VFA accumulation occurred only in Cycle 1 mono-digestion, while the adapted inoculum in Cycles 2 and 3 eased AD at low temperature, mitigating FAN inhibition. It underscores the potential of innovative approaches such as multi-substrate digestion, lowering temperature to overcome FAN inhibition, and mode of operation including bioaugmentation in maximizing biogas production efficiency under varying temperature conditions and substrate compositions.

## Conclusion

This study demonstrated multi-substrate AD at 20 ± 1 °C, using a two-stage solid-liquid system, achieving SMYs of 0.233 ± 0.028 L g^−1^ VS_fed_ (Set 1) and 0.262 ± 0.004 L g^−1^ VS_fed_ (Set 2) over a 68-day treatment period with an OLR of 4.5–5.1 g VS L^−1^ d^−1^ and a feed to inoculum ratio of 1:5–1:5.5. We provided a method for regular monitoring of system performance without compromising anaerobic conditions using stability ratios (e.g., TVFA/alkalinity) from the liquid digester. The ratio exceeding its stability threshold of 1.4 only during the mono-digestion of CM in Cycle 1 indicates better inoculum adaptation in Cycles 2 and 3. FAN concentrations in liquid digesters across cycles typically stayed below threshold levels, indicating the effectiveness of low-temperature AD. Thus, the development of solid inoculum from diluted liquid inoculum, its progressive adaptation during each treatment cycle, and its positive impact on overall AD, including reduced treatment periods at higher OLR, was successfully achieved. HM concentrations in the digestate were analyzed to reveal no bioaccumulation in the digestate post-AD which is one of the several crucial indicators of its biofertilizer potential. Further research on multi-substrate AD can support large-scale biogas systems for sustainable waste management practices in cold regions like Canada.

## Supplementary Information

Below is the link to the electronic supplementary material.

Supplementary file1 (PDF 197 KB)

## Data Availability

All data generated or analyzed during this study are included in this article.
