# Supplementary material for: A robust, low-temperature, closed-loop anaerobic system for high-solid mixed farm wastes: advancing agricultural waste management solutions in Canada
Source: Environ Sci Pollut Res Int. 2024 May 23;32(48):27841–59. doi: 10.1007/s11356-024-33654-7 (PMC12695939; doi:10.1007/s11356-024-33654-7)
Supplement: Supplementary file 1 — Supplementary file1 (PDF 197 KB) [file 11356_2024_33654_MOESM1_ESM.pdf]

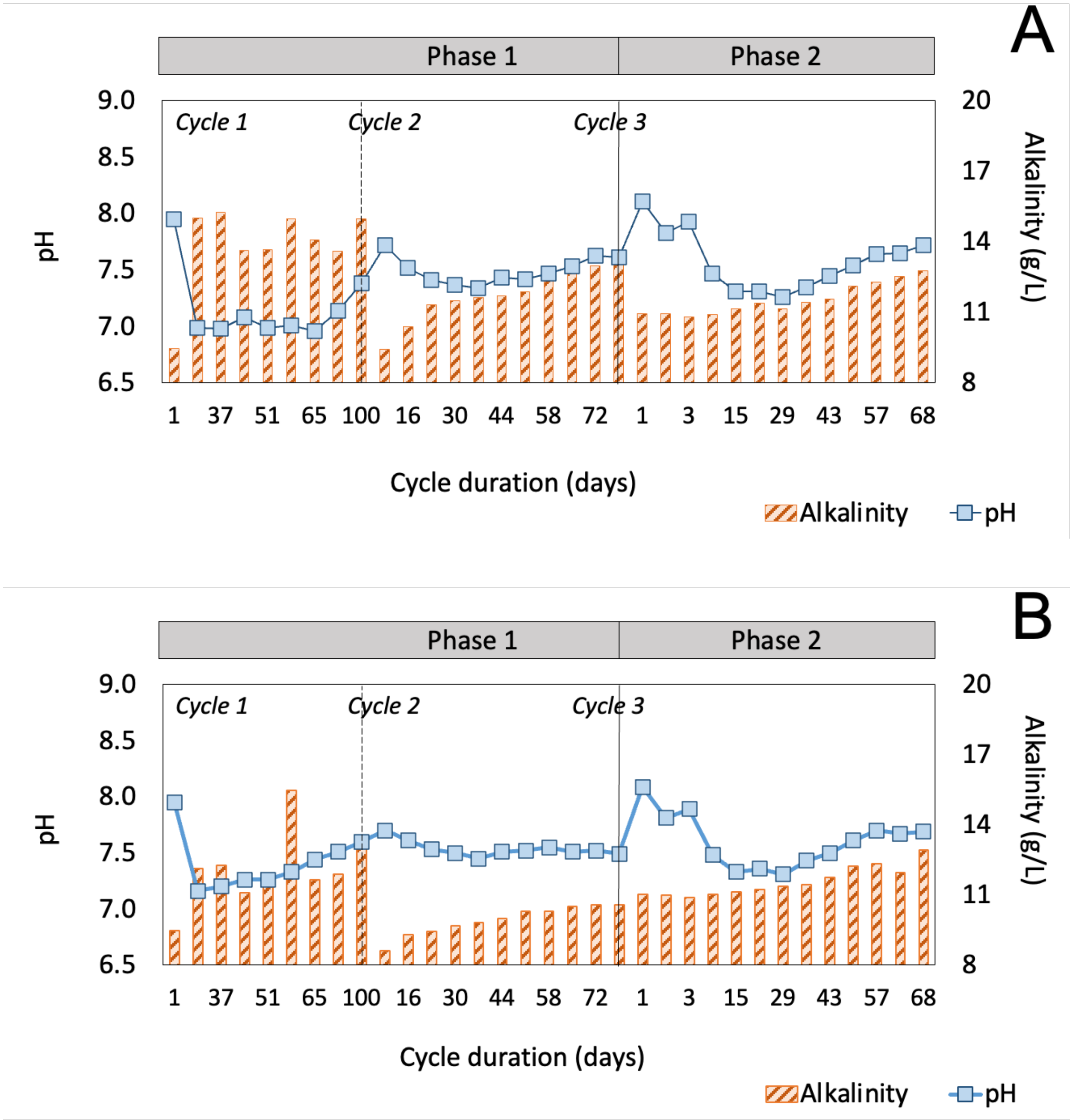

Figure S1 Evolution of pH and alkalinity observed in liquid digesters of Set 1 (A) and Set 2 (B) over three cycles
